# Serum Calretinin as a Biomarker in Malignant Mesothelioma

**DOI:** 10.3390/jcm10214875

**Published:** 2021-10-22

**Authors:** Cita Zupanc, Alenka Franko, Danijela Štrbac, Metoda Dodič Fikfak, Viljem Kovač, Vita Dolžan, Katja Goričar

**Affiliations:** 1 Military Medical Unit—Slovenian Army, 1000 Ljubljana, Slovenia; cita.zupanc@mors.si; 2 Faculty of Medicine, University of Ljubljana, 1000 Ljubljana, Slovenia; alenka.franko@kclj.si (A.F.); dstrbac@onko-i.si (D.Š.); metoda.dodicfikfak@kclj.si (M.D.F.); vkovac@onko-i.si (V.K.); 3 Clinical Institute of Occupational Medicine, University Medical Centre Ljubljana, 1000 Ljubljana, Slovenia; 4 Institute of Oncology Ljubljana, 1000 Ljubljana, Slovenia; 5 Pharmacogenetics Laboratory, Institute of Biochemistry and Molecular Genetics, Faculty of Medicine, University of Ljubljana, 1000 Ljubljana, Slovenia; vita.dolzan@mf.uni-lj.si

**Keywords:** malignant mesothelioma, calretinin, asbestos-related disease, biomarker

## Abstract

The early diagnosis of malignant mesothelioma (MM) could improve the prognosis of MM patients. To confirm an MM diagnosis, an immunohistochemical analysis of several tumor tissue markers, including calretinin, is currently required. Our aim is to evaluate serum calretinin as a potential biomarker in asbestos-related diseases, especially in MM. Our study includes 549 subjects: 164 MM patients, 117 subjects with asbestosis, 195 subjects with pleural plaques and 73 occupationally asbestos-exposed subjects without asbestos-related diseases. The serum calretinin concentration was determined with a commercially available enzyme immunoassay. Data on the soluble mesothelin-related peptides (SMRP) concentration are available from previous studies. MM patients had a significantly higher calretinin concentration than subjects without disease, subjects with pleural plaques or subjects with asbestosis (all *p* < 0.001). The histological type was significantly associated with serum calretinin: patients with sarcomatoid MM had lower calretinin than patients with the epithelioid type (*p* = 0.001). In a ROC curve analysis, the area under the curve for calretinin concentration predicting MM was 0.826 (95% CI = 0.782–0.869; *p* < 0.001). At the cutoff value of 0.32 ng/mL, sensitivity was 0.683, while specificity was 0.886. The combination of calretinin and SMRP had the highest predictive value. Calretinin is a useful biomarker that can distinguish MM from other asbestos-related diseases and could, therefore, contribute to an earlier non-invasive diagnosis of MM.

## 1. Introduction

Asbestos-related diseases represent a major public health problem all over the world [1,2,3]. Often, these are occupational diseases resulting from asbestos exposure in the workplace. Although the use and production of asbestos was legally banned in Slovenia in 1996, the burden of asbestos-related diseases has not yet been reduced, mostly due to the long latency period between asbestos exposure and disease development [4,5]. Asbestos-related diseases include pleural plaques, asbestosis, malignant mesothelioma (MM), lung cancer and other cancers.

MM is a rare but very aggressive tumor with a poor prognosis and a 5-year survival below 10% [6]. Early detection is crucial for better prognosis, but due to nonspecific symptoms, an MM diagnosis is usually determined only in the advanced stages [7,8]. The diagnosis of MM is confirmed pathohistologically by a panel of protein biomarkers determined on tumor tissue samples [8,9]. Positive immunohistochemical markers that can differentiate between malignant and benign mesothelial cells or other tumors with a high sensitivity and specificity include calretinin, cytokeratin 5/6 and WT1 for both pleural and peritoneal MM [10,11,12].

Several studies investigated the potential role of blood biomarkers in asbestos-related diseases, because this approach is less invasive than a tissue biopsy [13,14]. A number of soluble protein biomarkers that could contribute to the earlier diagnosis of MM and differentiate between MM and other asbestos-related diseases has been described. The most promising so far have been soluble mesothelin-related peptides (SMRP), fibulin, survivin, osteopontin and many others [15]. Additionally, they could also serve as prognostic biomarkers in MM and could help to evaluate tumor response to treatment [16]. However, due to the limited sensitivity or specificity, they are not used in clinical practice [16]. Studies suggest that a combination of biomarkers could improve the predictive values observed for individual biomarkers [16].

Recent studies tried to evaluate if the tissue MM biomarker calretinin could also be used as a soluble biomarker in MM [8,17,18,19]. Calretinin is a calcium-binding protein that plays an important role in neurons and is also expressed on the surface of mesothelial cells [17]. An increased calretinin expression has been associated with an increased proliferation, invasion and migration of mesothelial cells, as well as an epithelial-to-mesenchymal transition [18,19]. Studies suggest that calretinin may be involved in the cell response to asbestos fibers through the phosphatidylinositol 3-kinase (PI3K)/protein kinase B (AKT) signaling pathway [20]. Calretinin may also interact with focal adhesion kinase (FAK) and activate its signaling pathway that has often been associated with tumorigenesis [18].

In tumor samples, calretinin expression has been detected in up to 90% of MM patients with the epithelioid histological type, but only in up to 55% of patients with the sarcomatoid subtype [9]. In studies to date, the level of calretinin in tissue samples has been positively associated with the level of calretinin in plasma in patients with pleural MM [21]. A few recent studies showed that calretinin was increased also in the plasma or serum of MM patients compared to occupationally exposed subjects with other asbestos-related diseases or healthy controls [8,22,23,24]. Most studies focused on plasma calretinin, while only two studies investigated serum calretinin levels [8,22].

Our aim is to evaluate serum calretinin as a potential minimally invasive biomarker in asbestos-related diseases in a large Slovenian study group and to assess the association between clinical parameters and asbestos exposure and serum calretinin levels.

## 2. Materials and Methods

### 2.1. Study Population

In the study, we included MM patients, subjects with asbestosis, subjects with pleural plaques and occupationally asbestos-exposed subjects that did not develop any asbestos-related diseases.

Patients with MM were treated at the Institute of Oncology Ljubljana from November 2001 to March 2019. The diagnosis of pleural and peritoneal MM was performed by thoracoscopy or laparoscopy, respectively and confirmed histologically by an experienced pathologist. For pleural MM, stage was determined according to the TNM staging system. Performance status was determined using Eastern Cooperative Oncology Group (ECOG) scores.

Other subjects (subjects with asbestosis, subjects with pleural plaques, asbestos-exposed subjects who did not develop any asbestos-related diseases) were selected from a cohort of occupationally exposed workers who were evaluated by the State Board for the Recognition of Occupational Asbestos Diseases at the Clinical Institute of Occupational, Traffic and Sports Medicine in Ljubljana between 1 January 1999 and 31 December 2003. The diagnosis was based on the Helsinki Criteria for Diagnosis, Attribution of Asbestos Diseases [25] and the American Thoracic Society recommendations [26]. Follow-up confirming and updating the diagnosis was performed for all subjects in 2018.

Only individuals who confirmed their participation by signing the informed consent were included in the study. The study was approved by the National Medical Ethics Committee of the Republic of Slovenia (31/07/04, 39/04/06 and 41/02/09) and was carried out according to the Helsinki Declaration.

Demographic and clinical data were obtained from medical records or during an interview. Data on the cumulative asbestos exposure in fibers/cm^3^ years and the time of asbestos exposure were available for subjects with asbestosis, subjects with pleural plaques, subjects who did not develop any of asbestos related diseases and some subjects with MM. Data on cumulative asbestos exposure were calculated from individual time of exposure and corresponding exposure intensities as previously described [27,28]. For the subjects with available data on cumulative asbestos exposure, the asbestos exposure was categorized into three groups: low (<11 fibers/cm^3^ years), medium (11–20 fibers/cm^3^ years) and high (>20 fibers/cm^3^ years) asbestos exposure. For a subset of MM patients without information on cumulative asbestos exposure, the asbestos exposure was assessed based on precise work history obtained with a structured interview and comparison with exposures of the group of subjects with known cumulative asbestos exposure. Additionally, in these subjects, asbestos exposure was categorized as low, medium or high.

Serum samples for determination of protein biomarkers were collected at diagnosis for patients with MM and at inclusion in the study for all other subjects. Serum samples were prepared within 6 h of blood collection and stored at −20 °C until the analysis.

### 2.2. Measurement of Serum Calretinin

Serum calretinin levels were determined using a commercially available enzyme-linked immunosorbent assay (ELISA) for all subjects. We used the Calretinin ELISA assay (DLD Diagnostika GmbH, Hamburg, Germany), described in the literature [8,22], according to the manufacturer’s instructions. The manufacturer of the assay reported the intra-assay coefficient of variation (CV) for calretinin as 8.1–6.6% and inter-assay CV as 10.4–10.0%. The reported lower limit of detection was 0.05 ng/mL. Standard curve was calculated using a four-parameter logistic curve fit.

### 2.3. Measurement of Serum Soluble Mesothelin-Related Peptides

Data on serum soluble mesothelin-related peptides (SMRP) concentrations for a subset of subjects were available from our previous study, where we used a commercially available sandwich ELISA assay (MESOMARK^TM^, Fujirebio Europe BV, Breda, The Netherlands) [29].

### 2.4. Statistical Analysis

Continuous and categorical variables were described using median and 25–75% range or frequencies, respectively. Chi-square test was used to compare the distribution of categorical variables among different groups, while nonparametric Mann–Whitney test or Kruskal–Wallis test with post hoc Bonferroni corrections for pairwise comparisons were used for continuous variables. Correlations between continuous variables were calculated using Spearman’s rho coefficient. Receiver operating characteristic (ROC) curve analysis was used to determine area under the curve (AUC), sensitivity and specificity. Cut-off values were selected as the values with the highest sum of sensitivity and specificity. A multivariable logistic regression model was used to assess the combined effect of multiple variables and predictive scores were calculated based on regression coefficients. For the combination of calretinin and SMRP, the final model for the probability of occurrence of MM was: −3.854 + 4.561 × calretinin concentration + 0.539 × SMRP concentration. All statistical tests were two-sided and the level of significance was set at 0.05. The statistical analyses were carried out by using IBM SPSS Statistics version 27.0 (IBM Corporation, Armonk, NY, USA).

## 3. Results

Among 549 subjects included in our study, 164 (30%) had MM. Among 385 non-MM subjects that were occupationally exposed to asbestos, 73 did not develop any asbestos-related disease, 195 subjects had pleural plaques and 117 subjects had asbestosis. The characteristics of each subject group are presented in Table 1. The groups differed significantly by age; patients with MM were older than all other groups (*p* < 0.001).

**Figure 1:**
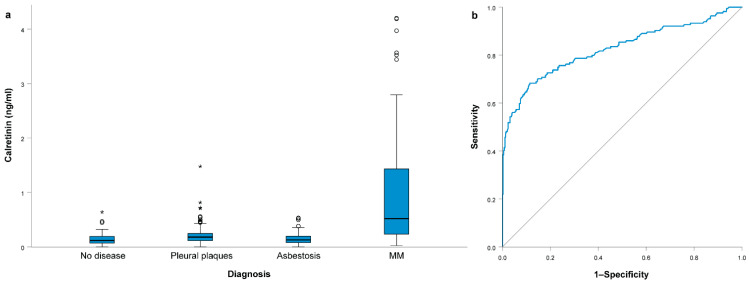
(**a**) Serum calretinin concentration in different subject groups; (**b**) ROC curve analysis for serum calretinin concentration comparing malignant mesothelioma (MM) patients with all other subjects. All boxplots represent median with interquartile range, while outliers are represented using circles and extreme outliers are represented using * symbol.

Serum calretinin concentration differed significantly among subject groups (*p* < 0.001); it was 0.52 (0.23–1.43) ng/mL in MM patients, 0.13 (0.08–0.2) ng/mL in subjects with asbestosis, 0.18 (0.12–0.25) ng/mL in subjects with pleural plaques and 0.12 (0.07–0.2) ng/mL in subjects without asbestos-related diseases (Figure 1a). After Bonferroni correction for multiple comparisons, patients with MM had a significantly higher calretinin concentration than subjects without disease (*p* < 0.001), subjects with pleural plaques (*p* < 0.001) and subjects with asbestosis (*p* < 0.001) (Figure 1a). The serum calretinin concentration was also slightly higher in subjects with pleural plaques than in subjects without any disease (*p* = 0.003) or asbestosis (*p* = 0.005).

In all subjects without MM, the calretinin concentration was 0.15 (0.09–0.23) ng/mL, significantly lower compared to patients with MM (*p* < 0.001). We performed an ROC curve analysis to compare patients with MM with other subjects (Figure 1b). The area under the curve (AUC) for calretinin concentration predicting MM was 0.826 (95% CI = 0.782–0.869; *p* < 0.001). At the cutoff value of 0.32 ng/mL, sensitivity for predicting MM was 0.683, while specificity was 0.886. For specificities of 0.900, 0.950, 0.970 and 0.990, sensitivities were 0.652, 0.561, 0.518 and 0.457, respectively, while calretinin cutoff values were 0.34 ng/mL, 0.45 ng/mL, 0.50 ng/mL and 0.64 ng/mL, respectively.

### 3.1. Association of Clinical Parameters with Calretinin Levels

**Figure 2:**
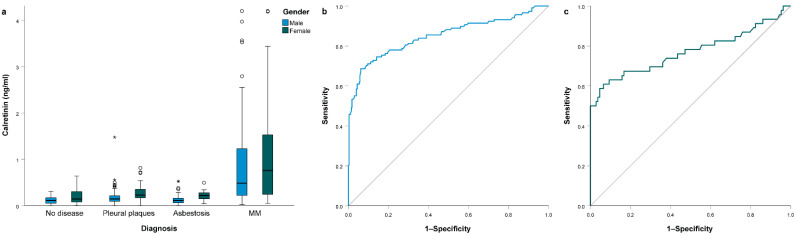
(**a**) Serum calretinin concentration in different subject groups stratified by gender; (**b**) ROC curve analysis for serum calretinin concentration comparing malignant mesothelioma (MM) patients with all other subjects among men; (**c**) ROC curve analysis for serum calretinin concentration comparing MM patients with all other subjects among women. All boxplots represent median with interquartile range, while outliers are represented using circles and extreme outliers are represented using * symbol.

Among the clinical parameters, gender was significantly associated with serum calretinin levels (Figure 2a). Women had higher values than men (*p* < 0.001). The difference in serum calretinin levels between women and men was significant in subjects without disease (*p* = 0.042), subjects with pleural plaques (*p* < 0.001) and subjects with asbestosis (*p* < 0.001). In patients with MM, even though women had higher values of calretinin concentration, the difference was not significant (*p* = 0.315) (Table S1). Additionally, older subjects in the whole study group had higher calretinin (*p* < 0.001), but no correlation was observed in individual study groups (all *p* > 0.05, Table S1). In subjects with pleural plaques, smokers had slightly lower calretinin (*p* = 0.019).

To account for gender differences, we also performed a separate ROC curve analysis in men and in women. In men, the AUC for calretinin concentration predicting MM was 0.850 (95% CI 0.802–0.899; *p* < 0.001). At the cutoff value of 0.32 ng/mL, sensitivity for predicting MM was 0.686, while specificity was 0.939 (Figure 2b). In women, AUC was 0.770 (95% CI 0.672–0.868; *p* < 0.001). At the cutoff value of 0.47 ng/mL, sensitivity for predicting MM was 0.609, while specificity was 0.935 (Figure 2c).

### 3.2. Association of Asbestos Exposure with Calretinin Levels

We also evaluated if asbestos exposure was associated with calretinin levels. Complete data on asbestos exposure were available for subjects without MM (Table S2). In subjects with asbestosis, a longer time of asbestos exposure was associated with slightly higher calretinin levels (Spearman’s rho = 0.187, *p* = 0.045, Table S3). A similar trend was observed in subjects with pleural plaques, but the correlation did not reach statistical significance (Spearman’s rho = 0.141, *p* = 0.057). On the other hand, cumulative asbestos exposure was not associated with calretinin levels (Table S3). In a group of MM patients with available data on asbestos exposure, no differences in calretinin levels were observed (Table S4).

### 3.3. Association of MM Clinical Parameters with Calretinin Levels

**Figure 3:**
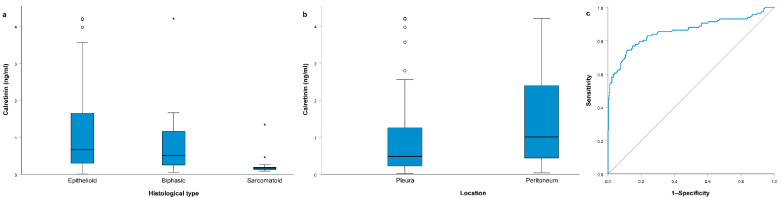
(**a**) Association of histological type with serum calretinin levels; (**b**) association of malignant mesothelioma (MM) location with serum calretinin levels; (**c**) ROC curve analysis for serum calretinin concentration comparing patients with epithelioid MM with all other subjects. All boxplots represent median with interquartile range, while outliers are represented using circles and extreme outliers are represented using * symbol.

Association of MM clinical parameters with calretinin levels is presented in Table 2. Among them, only the histological type was significantly associated with serum calretinin (*p* = 0.001, Figure 3a). The median calretinin concentration was 0.67 (0.30–1.66) ng/mL in the epithelioid type, 0.51 (0.20–1.17) ng/mL in biphasic and 0.17 (0.13–0.23) ng/mL in sarcomatoid MM. After correction for multiple comparisons, patients with sarcomatoid MM had significantly lower calretinin than patients with the epithelioid type (*p* = 0.001) and tended to have lower calretinin compared to patients with biphasic MM (*p* = 0.057). Patients with peritoneal MM tended to have even higher serum calretinin (1.00 (0.40–2.41) ng/mL) compared to patients with pleural MM (0.48 (0.22–1.27) ng/mL), but the difference did not reach statistical significance (*p* = 0.065, Figure 3b). There was no significant association between calretinin levels and the MM stage (*p* = 0.794) or Eastern Cooperative Oncology Group (ECOG) performance status (*p* = 0.191).

When only patients with epithelioid MM were compared to all subjects without MM in the ROC curve analysis, the AUC was 0.858 (95% CI 0.810–0.906; *p* < 0.001, Figure 3c). At the cutoff value of 0.32 ng/mL, sensitivity for predicting epithelioid MM was 0.744, while specificity was 0.886.

### 3.4. Combination of Soluble Mesothelin-Related Peptides and Calretinin

Data on both SMRP and calretinin levels were available in a subset of 416 patients: 68 MM patients, 106 subjects with asbestosis, 184 subjects with pleural plaques and 58 occupationally asbestos-exposed subjects that did not develop any asbestos-related diseases. Median SMRP levels at diagnosis were 0.25 (0–1.11) nmol/L in the entire group of subjects with available data; 0.17 (0–0.73) nmol/L among subjects without MM and 2.44 (0.51–9.73) nmol/L in patients with MM. SMRP and calretinin levels were significantly correlated in the whole study group (Spearman’s rho = 0.225, *p* < 0.001) and among patients with MM (Spearman’s rho = 0.443, *p* < 0.001).

A multivariable logistic regression model combining both calretinin and SMRP showed that, in our study, calretinin (OR = 95.67, 95% CI: 17.17–533.20, *p* < 0.001) was an even better predictor of MM than mesothelin (OR = 1.71, 95% CI: 1.34–2.20; *p* < 0.001).

**Figure 4:**
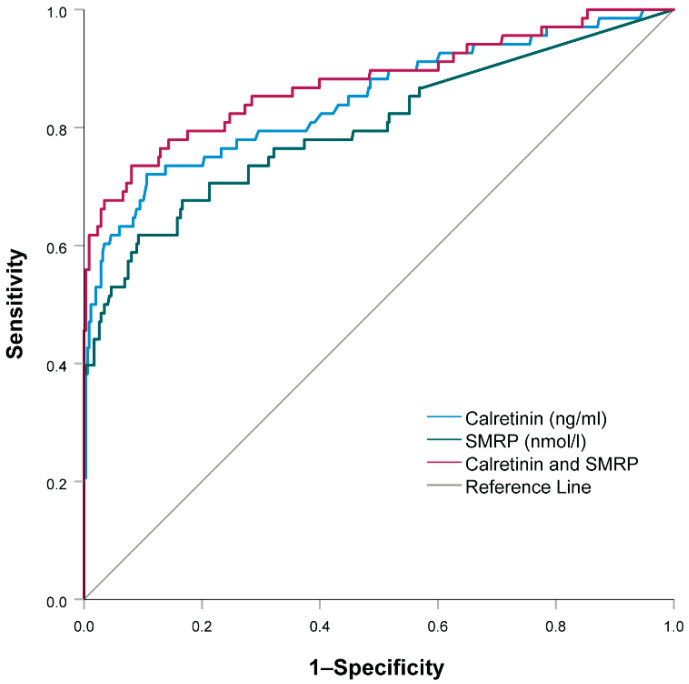
ROC curve analysis comparing serum calretinin, soluble mesothelin related peptides (SMRP) and their combination in malignant mesothelioma patients with all other subjects.

Using the ROC curve analysis, we also assessed how well calretinin or SMRP or both together could predict MM (Figure 4). The AUC for calretinin alone was 0.847 (95% CI = 0.787–0.907; *p* < 0.001). At the cutoff value of 0.32 ng/mL, sensitivity for predicting MM was 0.721, while specificity was 0.894. The AUC for SMRP alone was 0.800 (95% CI = 0.731–0.868). At the cutoff value of 1.55 nmol/L, sensitivity for predicting MM was 0.735, while specificity was 0.920. The AUC for the combination of calretinin and mesothelin was 0.877 (95% CI = 0.821–0.932; *p* < 0.001). At the cutoff value score of −1.69, sensitivity for predicting MM was 0.735, while specificity was 0.920. For specificities of 0.950, 0.970 and 0.990, sensitivities were 0.676, 0.662 and 0.618, respectively, while cutoff values were −1.32, −0.87 and −0.06, respectively.

## 4. Discussion

In the present study, we confirmed that serum calretinin was elevated in patients with MM and, thus, could serve as a potential minimally invasive diagnostic MM biomarker. Women had higher levels of calretinin compared to men, while the cumulative asbestos exposure did not affect calretinin levels. Calretinin was increased only in epithelioid and biphasic MM, but not in sarcomatoid MM. Although calretinin was an even better diagnostic biomarker of MM than SMRP, their combination had the highest sensitivity and specificity and, thus, the best predictive value for MM.

Calretinin values differed significantly among different subject groups included in our study. The highest values of calretinin were observed in subjects with MM compared to subjects with pleural plaques and asbestosis, and the lowest values in subjects without asbestos disease. All subjects without MM had similar serum calretinin, even though slightly higher values were observed for pleural plaques. Serum calretinin levels could successfully discriminate between MM patients and other asbestos-exposed subjects. At the cutoff value of 0.32 ng/mL, the specificity was high (89%), while the sensitivity was limited (68%). Consistent with our results, other studies to date have shown that patients with MM have higher levels of calretinin than other subjects who were also exposed to asbestos [8,21,30], suggesting calretinin behaves similarly to mesothelin and other biomarkers [30]. Similar plasma calretinin cutoff values were observed in previous studies [8,23], while higher cutoff values were required to reach 95% or 98% predefined specificity [8,30], similar to our study.

In the next part of the study, we assessed the influence of clinical parameters on calretinin concentration. We found that the concentration of calretinin was higher in women and a higher cutoff value was required to differentiate between MM patients and other subjects; however, the sensitivity was lower. This confirmed the results of other studies that also found higher levels of calretinin in small groups of women [21,24], except in one study evaluating plasma calretinin in a very small cohort of women from the Mexican population [23]. Other previous studies only evaluated calretinin levels in male subjects. This suggests separate cutoff calretinin values should be used for women and men when diagnosing MM.

In our study, older people had slightly higher levels of calretinin in the whole study group, but not within individual subject groups. This could be attributed to the fact that the patients with MM were older than other subjects. Additionally, smoking was not a predictor of calretinin levels.

According to our knowledge and available literature, we were the first to look closely at how asbestos exposure affects calretinin levels. A longer time of asbestos exposure had a statistically significant effect on higher calretinin levels only in subjects with asbestosis. On the other hand, we observed no effect of cumulative asbestos exposure or when patients were stratified according to low, medium or high asbestos exposure on calretinin levels. These results were consistent with a previous study where calretinin levels did not differ among subjects with moderate or high asbestos exposure [30].

We also compared the clinical parameters of MM with calretinin levels. We found that histological type of MM was associated with calretinin levels and significantly lower serum calretinin was observed in sarcomatoid MM. On the other hand, in our study, calretinin levels were not associated with the tumor stage and ECOG performance status. In epithelioid-type MM, calretinin had a better predictive value than in sarcomatoid-type MM, which was consistent with data from studies performed on tissue [9,31]. Previous studies, using a conventional case–control design, have shown that sarcomatoid MM is less well detected by the mesothelin and basically not detected by the calretinin assay [8,16], consistent with our results. The discovery of a biomarker that would be appropriate also for sarcomatoid MM is a major challenge for the future.

Most research in the field of MM biomarkers has been conducted only on the population of pleural MM [21,23]. In our study, we included patients with pleural and peritoneal MM. The results showed that patients with peritoneal MM tended to have even higher serum calretinin levels. These results were concordant with a comprehensive study of immunochemical markers in peritoneal MM, where calretinin was detected in all tissue samples and had the highest sensitivity [32]. Additionally, a set of five peritoneal MM cases included in the MoMar study also tended to have a higher median concentration of calretinin compared to cases with pleural MM [30]. However, additional studies on patients with peritoneal MM are needed to confirm our results.

In the last part of the study, we compared the diagnostic potential of calretinin and SMRP. SMRP has so far been considered as one of the best biomarkers for MM [16]. Our current study has shown that individually, calretinin was an even better diagnostic biomarker for MM. However, their combination had the highest sensitivity (73%) and specificity (92%). This finding was in agreement with the results of the MoMar Study Group and other studies [8,23,24,30]. The commercially available assays for calretinin and mesothelin approved for clinical diagnostics showed high specificities. Therefore, it would make sense to use a calretinin and SMRP combination as a diagnostic biomarker of MM. Additionally, a study found that the calretinin and mesothelin concentrations in MM cases appeared to increase mostly in the year before clinical diagnosis, suggesting they could be used as screening biomarkers in asbestos-exposed subjects [30].

In our study, calretinin and mesothelin were both measured in serum samples for a subset of subjects. In previous studies, mesothelin or SMRP were measured predominantly in serum [13,14,16,29]. On the other hand, studies looking at calretinin or calretinin and mesothelin combinations focused mostly on plasma levels [23,24,33,34]. A recent study suggested that an appropriate selection of serum or plasma is important for a reliable and repeatable biomarker detection for fibulin 3 [33]. On the other hand, different anticoagulants did not influence calretinin plasma levels [33]. In a previous study where calretinin was measured in serum and plasma, a difference between plasma and serum samples was not observed and the antigen showed a high stability [8]. Additionally, no significant differences between calretinin recovery rates in serum or plasma were detected and the storage time did not affect calretinin measurements [22]. Our results suggest that serum samples were appropriate for the detection of calretinin concentration.

Calretinin is involved in several important cell processes in MM and it has been associated with the transition of mesothelial cells to tumor cells [17]. As its downregulation decreases cell growth and viability and has been associated with an increased intrinsic apoptosis pathway, it has also been proposed as a potential new target in MM; however, more studies are needed in this field [19,20]. Additionally, further studies are needed to elucidate the source of calretinin in body fluids. In previous studies, calretinin was already detected in extracellular vesicles (EVs) derived from MM cell lines and in pleural effusions [35,36], which suggests EVs could contribute to calretinin transfer. Future studies evaluating calretinin in EVs from serum or plasma of MM patients could help determine if EV-associated calretinin could be a suitable biomarker for MM or help identify other biomarkers used in combination with calretinin.

According to our knowledge, our study was the first to perform a comprehensive evaluation of serum calretinin levels in an independent population. The advantage of our study was that we measured the value of calretinin in several different subject groups (no disease, asbestosis, pleural plaques and MM) and that we were able to evaluate gender differences, the role of asbestos exposure and MM clinical parameters. On the other hand, a limitation of our study was that asbestos exposure and SMRP data were not available for all subjects. Asbestos exposure was evaluated as cumulative asbestos exposure; however, the data on the number of asbestos bodies were not available. In our study, we also did not evaluate the calretinin concentration in healthy individuals without an occupational asbestos exposure. However, previous studies performed in healthy individuals without malignant disease enrolled from a general population suggest calretinin concentrations in the population are similar to concentrations observed in healthy occupationally asbestos-exposed subjects and are not only associated with asbestos exposure [34,37].

Several factors that could affect the concentration of calretinin and mesothelin were investigated in previous studies in healthy male subjects [34,37]. The concentrations of calretinin and mesothelin increased with increased cystatin C reflecting renal dysfunction. Mesothelin was additionally affected by bronchitis, elevated C-reactive protein and current hypertension [34]. Apart from gender, other factors could, therefore, influence the determination of appropriate cutoff values for calretinin. However, no data on renal function were available in our study. These results should be confirmed in further studies in asbestos-exposed subjects. Additionally, MM patients and other subjects in our study were not age-matched. However, previous studies suggest age did not affect the calretinin concentration [8,24].

In conclusion, our study confirmed that serum calretinin could serve as a diagnostic marker differentiating between MM and other asbestos-related diseases or asbestos-exposed controls without asbestos-related diseases. The results could contribute to an earlier diagnosis and better prognosis of MM and to a better understanding of various asbestos-related diseases.

## Figures and Tables

**Table 1:** Clinical characteristics of the subjects included in the study.

Characteristic, Category/Unit	No Disease (N = 73)	Pleural Plaques (N = 195)	Asbestosis (N = 117)	MM (N = 164)	*p*
Gender					
Male, N (%)	52 (71.2)	134 (68.7)	91 (77.8)	118 (72.0)	0.392 ^a^
Female, N (%)	21 (28.8)	61 (31.3)	26 (22.2)	46 (28.0)	
Age					
Years, Median (25–75%)	53.4 (47.7–60.7)	55.1 (48.8–64)	59.7 (51.1–66.6)	66.5 (60–73)	<0.001 ^b^
Smoking					
No, N (%)	38 (52.1)	99 (50.8)	56 (47.9)	97 (61.0) [5]	0.125 ^a^
Yes, N (%)	35 (47.9)	96 (49.2)	61 (52.1)	62 (39.0)	

^a^ Comparison of all four subject groups calculated using chi-square test; ^b^ comparison of all four subject groups calculated using Kruskal–Wallis test. MM, malignant mesothelioma. Number of missing data is presented in [] brackets.

**Table 2:** Association of malignant mesothelioma (MM) clinical parameters with calretinin levels.

Characteristic	Category	N (%)	Calretinin Level (ng/mL) Median (25–75%)	*p*
Histological type	Epithelioid	117 (71.3)	0.67 (0.30–1.66)	0.001 sarcomatoid vs. epithelioid: *p* = 0.001 sarcomatoid vs. biphasic: *p* = 0.057
	Biphasic	20 (12.2)	0.51 (0.20–1.17)	
	Sarcomatoid	13 (7.9)	0.17 (0.13–0.23)	
	Not determined	14 (8.5)	0.44 (0.20–0.65) ^a^	
Stage	1	8 (4.9) [1] ^b^	0.36 (0.17–0.93)	0.794
	2	37 (22.7)	0.44 (0.26–1.02)	
	3	48 (29.4)	0.56 (0.22–1.43)	
	4	50 (30.7)	0.50 (0.21–1.70)	
Location	Pleural MM	144 (87.9	0.48 (0.22–1.27)	0.065
	Peritoneal MM	20 (12.2)	1.00 (0.40–2.41)	
ECOG	0	8 (4.9) [1]	0.32 (0.08–1.08)	0.191
	1	81 (49.7)	0.48 (0.21–1.17)	
	2	64 (39.3)	0.68 (0.24–1.67)	
	3	10 (6.1)	0.50 (0.39–0.99)	

^a^ Patients with undetermined histological type were not included in further comparisons; ^b^ stage was determined only in patients with pleural MM. ECOG, Eastern Cooperative Oncology Group performance status. Number of missing data is presented in [] brackets.

## Data Availability

All the data are presented within the article and in the Supplementary Material. Any additional information is available on request from the corresponding author.

## Supplementary Materials

The following are available online at www.mdpi.com/article/10.3390/jcm10214875/s1, Table S1: association of clinical parameters with serum calretinin levels, Table S2: data on asbestos exposure among subjects without malignant mesothelioma, occupationally exposed to asbestos, Table S3: association of asbestos exposure with calretinin levels among subjects without malignant mesothelioma, occupationally exposed to asbestos, Table S4: association of asbestos exposure with calretinin levels among patients with malignant mesothelioma.
