# Supplementary material for: Serum Calretinin as a Biomarker in Malignant Mesothelioma
Source: J Clin Med. 2021 Oct 22;10(21):4875. doi: 10.3390/jcm10214875 (PMC8585060; doi:10.3390/jcm10214875)
Supplement: Supplementary file 1 [file jcm-10-04875-s001.zip › jcm-1390731-supplementary.pdf]

Table S1: Association of clinical parameters with serum calretinin levels.

		All subjects		No disease (N=73)		Pleural plaques (N=195)		Asbestosis (N=117)		MM (N=164)	
**Characteristic**	**Category/Unit**	**Median calretinin (25%-75%)***	**P**	**Median calretinin (25%-75%)***	**P**	**Median calretinin (25%-75%)***	**P**	**Median calretinin (25%-75%)***	**P**	**Median calretinin (25%-75%)***	**P**
Gender	Male	0.16 (0.09-0.32)	<0.001	0.11 (0.06-0.17)	0.042	0.15 (0.10-0.21)	<0.001	0.11 (0.06-0.16)	<0.001	0.48 (0.22-1.24)	0.315
	Female	0.25 (0.15-0.46)		0.15 (0.08-0.31)		0.23 (0.18-0.35)		0.22 (0.15-0.28)		0.76 (0.24-1.56)	
Smoking	No	0.21 (0.12-0.42)	<0.001	0.12 (0.07-0.26)	0.183	0.19 (0.14-0.28)	0.019	0.15 (0.09-0.23)	0.167	0.51 (0.25-1.33)	0.802
	Yes	0.16 (0.09-0.29)		0.12 (0.05-0.17)		0.16 (0.11-0.23)		0.12 (0.06-0.19)		0.53 (0.18-1.45)	
		**Spearman's rho**	**P**	**Spearman's rho**	**P**	**Spearman's rho**	**P**	**Spearman's rho**	**P**	**Spearman's rho**	**P**
Age	Years	0.310	<0.001	0.034	0.773	0.132	0.066	0.163	0.079	0.051	0.520

*calretinin levels in ng/ml
MM: malignant mesothelioma

Table S2: Data on asbestos exposure among subjects without malignant mesothelioma, occupationally exposed to asbestos.

Characteristic	Category/Unit	All subjects (N=385)	No disease (N=73)	Pleural plaques (N=195)	Asbestosis (N=117)
Asbestos exposure	Low, N (%)	257 (67.3) [3]	58 (79.5)	140 (72.5) [2]	59 (50.9) [1]
	Middle, N (%)	49 (12.8)	6 (8.2)	22 (11.4)	21 (18.1)
	High, N (%)	76 (19.9)	9 (12.3)	31 (16.1)	36 (31.0)
Cumulative asbestos exposure	Fibres/cm^3-years, Median (25%-75%)	4.7 (1.9-15.0) [3]	2 (0.6-5.8)	3.9 (1.9-11.8) [2]	10.3 (4.3-23.2) [1]
Time of asbestos exposure	Months, Median (25%-75%)	270 (148-343) [14]	259 (104.5-337)	264.5 (105-339) [13]	294 (204-356.5) [1]

Number of missing data is presented in [] brackets.

Table S3: Association of asbestos exposure with calretinin levels among subjects without malignant mesothelioma, occupationally exposed to asbestos.

		All subjects		No disease		Pleural plaques		Asbestosis	
**Characteristic**	**Category/Unit**	**Median calretinin (25%-75%)***	**P**	**Median calretinin (25%-75%)***	**P**	**Median calretinin (25%-75%)***	**P**	**Median calretinin (25%-75%)***	**P**
Asbestos exposure	Low	0.15 (0.09-0.22)	0.727	0.12 (0.07-0.21)	0.381	0.17 (0.11-0.24)	0.527	0.13 (0.08-0.19)	0.688
	Middle	0.15 (0.08-0.23)		0.09 (0.04-0.12)		0.20 (0.13-0.29)		0.14 (0.04-0.19)	
	High	0.15 (0.10-0.25)		0.08 (0.06-0.22)		0.19 (0.12-0.25)		0.13 (0.10-0.24)	
		**Spearman's rho**	**P**	**Spearman's rho**	**P**	**Spearman's rho**	**P**	**Spearman's rho**	**P**
Cumulative asbestos exposure	Fibres/cm^3-years	-0.025	0.631	0.005	0.966	0.053	0.460	0.118	0.208
Time of asbestos exposure	Months	0.070	0.178	-0.143	0.228	0.141	0.057	0.187	0.045

*calretinin levels in ng/ml

Table S4: Association of asbestos exposure with calretinin levels among patients with malignant mesothelioma.

Asbestos exposure	N (%)	Calretinin level (ng/ml) Median (25%-75%)	P
Low	19 (40.4)	0.44 (0.22-2.47)	0.350
Middle	13 (27.7)	0.37 (0.24-1.66)	
High	15 (31.9)	0.48 (0.12-1.18)	
